# Clinical course of 63 patients with neonatal onset urea cycle disorders in the years 2001–2013

**DOI:** 10.1186/s13023-016-0493-0

**Published:** 2016-08-19

**Authors:** Caroline Unsinn, Anibh Das, Vassili Valayannopoulos, Eva Thimm, Skadi Beblo, Alberto Burlina, Vassiliki Konstantopoulou, Sebene Mayorandan, Pascale de Lonlay, Jörg Rennecke, Jens Derbinski, Georg F. Hoffmann, Johannes Häberle

**Affiliations:** 1 Division of Metabolism and Children’s Research Center, University Children’s Hospital Zurich, 8032 Zürich, Switzerland; 2 Department of Paediatrics, Medical School Hannover, Hannover, Germany; 3 Reference Center for Inherited Metabolic Disorders, Necker-Enfants Malades University Hospital and IMAGINE Institute, Paris, France; 4 University Children’s Hospital, Düsseldorf, Germany; 5 Hospital for Children and Adolescents, Centre of Paediatric Research (CPR), University of Leipzig, Leipzig, Germany; 6 Department of Pediatrics University Hospital, Division of Inherited Metabolic Disorders, Padova, Italy; 7 University Children’s Hospital, Vienna, Austria; 8 Cytonet GmbH & Co. KG, Weinheim, Germany; 9 Department of Pediatrics, University Hospital Heidelberg, Heidelberg, Germany

**Keywords:** Urea cycle disorders, neonatal hyperammonemia, hyperammonemic crisis, Carbamoyl phosphate synthetase 1 deficiency, Ornithine transcarbamylase deficiency, Argininosuccinate synthetase deficiency, Dialysis

## Abstract

**Background:**

Urea cycle disorders (UCDs) are rare inherited metabolic defects of ammonia detoxification. In about half of patients presenting with a UCD, the first symptoms appear within a few days after birth. These neonatal onset patients generally have a severe defect of urea cycle function and their survival and outcome prognoses are often limited.

To understand better the current situation of neonatal onset in UCDs, we have performed a multicentre, retrospective, non-interventional case series study focussing on the most severe UCDs, namely defects of carbamoyl phosphate synthetase 1 (CPS1), ornithine transcarbamylase (OTC), and argininosuccinate synthetase (ASS).

**Methods and results:**

Data of 63 patients were collected (27 patients with ASS deficiency, 23 patients with OTC deficiency, and 12 patients with CPS1 deficiency, one patient definite diagnosis not documented). The majority of patients (43/63, 68 %) had an initial ammonia concentration exceeding 500 μmol/L (normal < 100), of which most (26/43, 60.5 %) were also encephalopathic and were treated with hemodialysis. In patients surviving the initial crisis, recurrence of hyperammonemic events within the first 1.5 years of life occurred frequently (mean 3.6 events, range 0–20). Of all patients, 16 (25.4 %) died during or immediately after the neonatal period.

**Conclusion:**

We observed in this cohort of neonatal onset UCD patients a high rate of initial life-threatening hyperammonemia and a high risk of recurrence of severe hyperammonemic crises. These corresponded to a high mortality rate during the entire study period (30.2 %) despite the fact that patients were treated in leading European metabolic centers. This underlines the need to critically re-evaluate the current treatment strategies in these patients.

## Background

The urea cycle is the main pathway for detoxification of nitrogenous waste products to urea and for arginine synthesis in the human body. Urea cycle disorders (UCDs) are due to enzyme or transporter defects in this metabolic pathway. UCD patients are prone to metabolic decompensation with severe hyperammonemia, which can result in brain edema and even death if treatment is initiated too late [1, 2]. UCDs are panethnic with an overall incidence, including late onset forms, of about 1 in 35.000 [3] but this can vary in different populations [4].

The first presentation of a UCD can be at any age in life and several different factors can trigger the metabolic crisis [5, 6]. Approximately half of the patients present in the first days of life after a short latent period [2, 7, 8]. In these patients, the physiological catabolism of the newborn is considered to trigger the decompensation. It is reasonable to assume that especially severe UCDs with (almost) complete loss of function are prone to this early onset. In contrast, patients with first symptoms beyond the neonatal period are classified as late onset UCDs and for these patients a variety of triggering factors are known, mainly common febrile infections, protein loading, trauma and other catabolic situations [9, 10].

In UCDs, insufficient detoxification of ammonia with subsequent accumulation in the entire body but mainly in the brain is the common pathophysiological mechanism. Ammonia is a neurotoxic substance, which explains why most clinical sequelae affect the central nervous system [1, 11]. The mechanisms of neurotoxicity are not entirely elucidated but it is clear that ammonia has several deleterious effects on brain cells, including swelling of astrocytes via glutamine accumulation, mitochondrial dysfunction, and neurotransmitter dysbalance [12–15].

Ammonia neurotoxicity becomes irreversible after a short interval (sometimes only few hours), especially if ammonia concentrations are highly elevated [16]. The duration of coma and the extent of ammonia elevation are the most important prognostic factors for patients in hyperammonemic crises [17, 18]. Although there are single patients that survive such episodes, the prognosis *quoad vitam* is commonly considered to be poor in cases of an ammonia level ≥ 1000 μmol/L (reference range ≤ 100 in newborns and ≤ 50 beyond the neonatal period) and/or a duration of hyperammonemic crisis of > 24 h [6].

Several studies describing cohorts treated before the year 2000 report a poor overall prognosis for UCD patients [19, 20], especially those with a neonatal onset of disease. However, the observations in these studies are of limited value for counselling current patients and families, as management modalities have improved. More recent reports indicate improved patient outcome with respect to the likelihood of survival for neonatal onset patients [7, 21]. This improvement has been hypothesized to be due to earlier diagnosis and better treatments [7].

Since data on the current prognosis of neonatal onset UCD patients are still scarce and often derived from only small cohorts, we collected information on UCD patients treated after the year 2001 in leading European centers. We performed a multicentre, retrospective, non-interventional case series study of neonatal onset UCD patients with an observation period of up to five years or until death. We focused on UCDs where the presentation was more likely to be severe and in the neonatal period: carbamoyl phosphate synthetase 1 (CPS1, OMIM *608307) deficiency (CPS1D, OMIM #23730), ornithine transcarbamylase (OTC, OMIM *300461) deficiency (OTCD, OMIM #311250, and argininosuccinate synthetase (ASS, OMIM *603470) deficiency (ASSD, OMIM #215700). The aim of this study was an improved understanding of the current situation of neonatal onset UCD patients and to identify aspects in the management of those patients that are relevant for future studies.

## Methods

### Study population

To characterise the clinical course of neonatal onset UCDs, we performed a multicentre, retrospective, non-interventional study collecting data on patients affected by symptomatic hyperammonemia in the neonatal period and treated according to local protocols in the years 2001 until 2013. Inclusion criteria were: neonatal onset CPS1, OTC or ASS deficiencies with enzymatic or molecular genetic confirmation, demographic data and medical history, and data until death, orthotopic liver transplantation (OLT), or at least six months of follow-up. Exclusion criteria were: birth before 2001, severe chronic or systemic disease other than UCD, and participation in the liver cell transplantation study SELICA (Safety & Efficacy of Liver Cell Application, Cytonet, Weinheim, Germany). The observation period of patients according to the study protocol was up to five years or until death, but the analysis period was only 550 days. We decided for this shorter analysis period since data was documented beyond 550 days in so few cases, rendering analysis beyond this period uninformative. The decision for this shortened analysis period was made before data evaluation.

### Choice of study sites

Patients were included from eight metabolic tertiary centres in five different European countries in order to avoid bias resulting from single centre observations. Study sites were chosen based on the presence of permanent senior metabolic specialists and on the availability of written management protocols that were, however, subject to local variability and may have changed during the study period.

### Data collection, ethics, consent and permissions

Study sites identified records from all UCD patients treated in the years 2001 to 2013. Patients with CPS1, OTC and ASS deficiencies were included if the observation period was at least six months or until death or OLT. Documentation of the patients included information on basic clinical and biochemical parameters, hyperammonemic events (HE, defined as plasma ammonia levels ≥ 150 μmol/L together with clinical symptoms probably related to hyperammonemia), medication, diet, and clinical outcome.

Parameters evaluated for this study included the number, severity and duration of HEs, concentrations of plasma ammonia and glutamine levels, and, if applicable, age in months at the time of death or at the time of OLT.

Primary endpoints were the time to the first HE after the initial HE, the number and duration of HEs, and peak plasma ammonia concentrations; events of increased plasma glutamine (= “hyperglutaminemic events”), defined as glutamine levels ≥ 1000 μmol/L (reference ranges for glutamine varied between laboratories and are age-dependent), time to the first hyperglutaminemic event, and peak plasma glutamine concentrations; hospitalizations due to HE; survival for at least six months.

Secondary endpoints were details of medication and other treatment modalities; time and outcome of OLT; intake of protein.

Data collection was initiated after approval by the local institutional review board was obtained. Since all data collected in this study came from patient charts at sites, and since this study was observational and non-interventional in design and only anonymized data were transferred to the sponsor, it was justified by the local authorities not to obtain informed consent from patients and parents. The only exception was in France where it is a legal requirement to seek individual approval before conducting any data processing.

### Statistical analyses

Data were analysed as indicated using descriptive statistics and Kaplan-Meier plots. A Microsoft Windows Server 2003 operating system and several working stations with Microsoft Windows XP Professional operating system were used for data management and statistical analyses. SAS 9.2 and SAS 9.3 for Microsoft Windows were the statistical packages in use. All programming of tables, figures, listings, and statistical analyses were performed using the statistical software package SAS® versions 9.2 and 9.3. After preparation of figures with SAS, the graphical presentations were produced using alternative software packages (e.g. EXCEL).

## Results

### Study population and baseline characteristics

The individual retrospective observation and data collection period in this study was until death, OLT, or at least 6 months (maximum five years) but the analysis period was limited to 550 days. The first and last dates documented were February 2001 and February 2013, respectively.

**Fig. 1:**
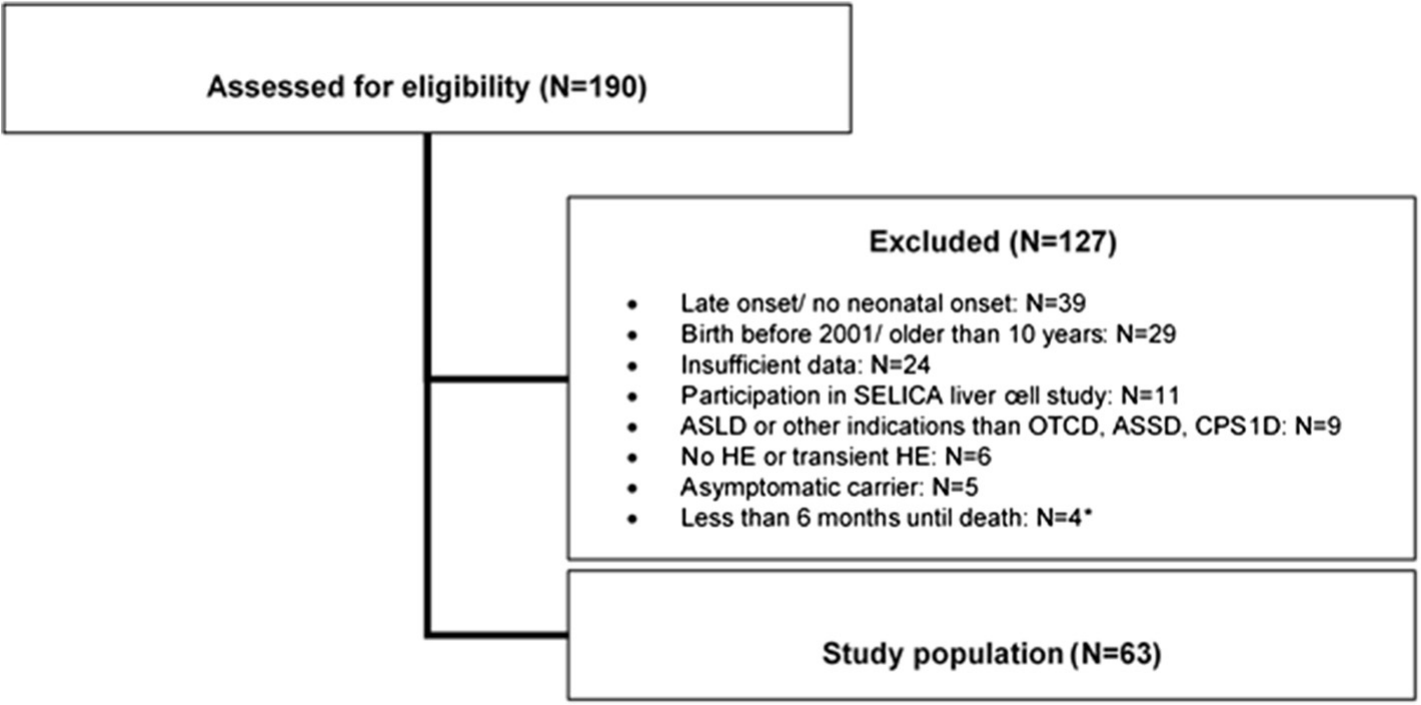
Flow chart of screened patients with exclusion criteria and final size of study population. ASSD: Argininosuccinate synthetase deficiency, ASLD: Argininosuccinate lyase deficiency, CPS1D: Carbamoyl phosphate synthetase 1 deficiency, HE: Hyperammonemic event, N: Number, OTCD: Ornithine transcarbamylase deficiency, SELICA: Safety & Efficacy of Liver Cell Application, study on liver cell transplantation as treatment option for severe metabolic disorders in patients with UCDs. *These patients would have been eligible for this study but were only recorded on the screening log and not documented in the database

In total, 190 patients from the eight study centres were assessed for eligibility. After exclusion of 127 patients based on different reasons (reasons for exclusion of patients are given in Fig. 1), 63 patients remained as the study population and were further analysed. The screening logs indicated that participating sites went thoroughly through their patient records and indeed considered all patients potentially eligible for inclusion into the study. Nevertheless, it cannot be excluded that some patients were missed. Analysis of screening logs further revealed that four patients who died early would have been eligible for the study but were recorded only on the screening log with no documentation in the database.

Within the study population, the most frequent diagnosis was ASSD accounting for 27 of 63 patients (42.8 %), followed by OTCD for 23 patients (36.5 %), and CPS1D for 12 patients (19 %). In one patient, no definite diagnosis was documented in the study records; nevertheless, to avoid data bias, this patient remained included in this study (Table 1).

**Table 1:** Baseline characteristics of the study population, diagnosis and gender

	CPS1D	OTCD	ASSD	Study population
Patients	12	23	27	63^a^
Gender				
male	4 (36.4 %)	21 (91.3 %)	12 (44.4 %)	37 (59.7 %)
female	7 (63.6 %)	2 (8.7 %)	15 (55.6 %)	25 (40.3 %)
no gender recorded	1			1

*ASSD* Argininosuccinate synthetase deficiency, *CPS1D* Carbamoyl phosphate synthetase 1 deficiency, *OTCD* Ornithine transcarbamylase deficiency

^a^one patient missing the documentation of the diagnosis

Out of the 23 OTC deficiency patients, 21 (91.3 %) were male, resulting in a total of about 60 % male patients included in the study, despite the fact that slightly more female than male patients were included with CPS1 and ASS deficiencies (63.6 and 55.6 %, respectively).

### Hyperammonemic events

During the initial metabolic crisis, the mean peak ammonia concentration was 1378 μmol/L (SD 950 μmol/L, range 161–4050) and the mean duration of the hyperammonemic crisis was five days (range 1–19). Of all included patients, 84.3 % experienced an initial ammonia concentration > 500 μmol/L and the initial crisis lasted for the majority of patients (*n* = 34; 66.7 %) two to seven days. Nine patients did not experience an ammonia level > 150 μmol/L as they were treated prospectively based on family history and/or prenatal diagnosis (details in Table 2).

**Table 2:** Characteristics of initial hyperammonemic episodes in all patients

	CPS1D	OTCD	ASSD	All patients
Number of patients	12	23	27	63
First hyperammonemic crisis ≥ 150 μmol/L		*N* = 20^a^		*N* = 60^a^
No	–	3 (15.0 %)	6 (22.2 %)	9 (15.0 %)
Yes	12 (100.0 %)	17 (85.0 %)	21 (77.8 %)	51 (85.0 %)
Peak concentration [μmol/L]				
Minimum	203	385	161	161
Maximum	2627	4050	2690	4050
Mean	1257	1875	1084	1378
SD	929	1105	682	950
150 – < 250	1 (8.3 %)	–	1 (4.8 %)	2 (3.9 %)
250 – < 500	2 (16.7 %)	1 (5.9 %)	3 (14.3 %)	6 (11.8 %)
≥ 500	9 (75.0 %)	16 (94.1 %)	17 (81.0 %)	43 (84.3 %)
Duration of crisis				
1 day	–	3 (17.6 %)	3 (14.3 %)	6 (11.8 %)
2–7 days	8 (66.7 %)	9 (52.9 %)	16 (76.2 %)	34 (66.7 %)
8–14 days	3 (25.0 %)	4 (23.5 %)	2 (9.5 %)	9 (17.6 %)
15–19 days	1 (8.3 %)	1 (5.9 %)	–	2 (3.9 %)

Peak concentration of ammonia is the highest level of plasma ammonia during the initial crisis. *SD* Standard deviation

^a^three missing values

Of the 43 patients with an initial HE > 500 μmol/L (Table 2), the majority were encephalopathic and required dialysis (*n* = 26; 60.5 %). Within this group, almost all (42/43) were treated in parallel with infusions of nitrogen scavengers and/or amino acids. Patients with a less severe initial HE (ammonia 150–500 μmol/L) rarely developed encephalopathy (only 1/8), and were mainly treated with nitrogen scavengers alone (6/8) but not with dialysis.

During the analysis period of a maximum of 550 days or until OLT or death, after their initial metabolic crisis patients suffered a mean of 3.6 HE (SD 4.8, range 0–20). The mean peak plasma ammonia level during these HEs was 539 μmol/L (SD 391, range 88–2060). Again, the majority of these events (71.8 %) lasted for two to seven days; whereby eight HEs (20.5 %) were limited to a single day, and only three HEs (7.7 %) lasted > 8 days. HEs that occurred after the initial crisis were generally less severe (in terms of extent and duration of hyperammonemia) than the first episode.

In 44 patients, HEs with ammonia levels ≥ 500 μmol/L were recorded. Of those 44 patients, 26 patients survived one (*n* = 20), two (*n* = 4), or three (*n* = 2) of the events. Of the 18 patients who died during the HE or in the later course of the study, 13 patients died immediately during the first (*n* = 12) or second (*n* = 1) event. Only single patients had initially survived one (*n* = 3), three (*n* = 1), or four (*n* = 1) episodes but died during later course of the study (Table 3).

**Table 3:** Incidences of hyperammonemic events with a peak ammonia ≥ 500 μmol/L, maximum analysis period 550 days

Parameter	CPS1D	OTCD	ASSD	All patients
Number of patients	12	23	27	63
Type of hyperammonemic event				
▪ no event ≥ 500 μmol/L	3	6	10	19
▪ at least one event ≥ 500 μmol/L, patient alive	5	7	13	26
individual number of events ≥ 500 μmol/L				
1	3	6	10	20
2	1	1	2	4
3	1	–	1	2
▪ at least one event ≥ 500 μmol/L, patient died	4	10	4	18
individual number of events ≥ 500 μmol/L				
1	4	9	2	15
2	–	1	–	1
3	–	–	1	1
4	–	–	1	1

**Fig. 2:**
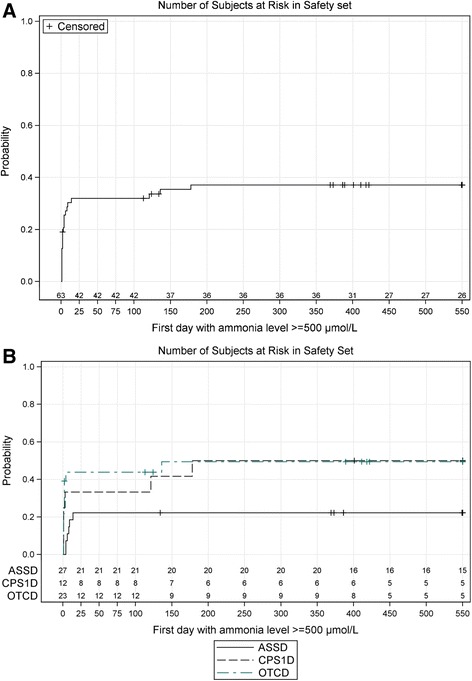
**a** Kaplan Meier plot of the time (shown in days of life of the patients) to first occurrence of ammonia ≥ 500 μmol/L during or after the initial crisis in all patients. This figure includes all 12 patients that died during the initial crisis. **b** Kaplan Maier plot of the time (shown in days of life of the patients) to first occurrence of ammonia ≥ 500 μmol/L, separated by the three groups of patients (ASSD, CPS1D, OTCD)

The likelihood of having a HE ≥ 500 μmol/L is highest in the first 28 days in all patient groups (Fig. 2a), and an additional 37.2 % ± 6.2 % risk for a further crisis until day 550. Patients with OTCD and CPS1D suffer these HEs earlier and with a higher probability in the first 25 days than patients with ASSD (Fig. 2b). When the incidences of HEs were calculated per 30 days, there was a significant difference between the OTCD or CPS1D and ASSD groups (*U*-test OTCD versus ASSD: *p* = 0.0117 and CPS1D versus ASSD: *p* = 0.0388) but not between the proximal UCDs (*U*-test OTCD versus CPS1D: *p* = 0.7176). 13 patients had their first HE during the neonatal period with ammonia > 500 μmol/L, but no further HEs for at least 180 days; one additional patient, diagnosed through positive family history and early treated, did not have an initial ammonia level > 500 μmol/L and no further HE for 180 days (Fig. 2a, b).

### Plasma glutamine concentrations

Plasma glutamine was recorded through the study from a total of 58 patients, as values from five patients were missing. Patients suffered a mean of 2.7 ± 3.7 (range 0–19) events with glutamine concentrations ≥ 1000 μmol/L (reference ranges for glutamine varied between laboratories and are age-dependent; the upper limit of normal is between 746 and 798 μmol/L according to [22]) over the analysis period. The mean peak glutamine level was 1400 ± 662 μmol/L (range 680–5428). The hyperglutaminemic events lasted in 26 patients (48.1 %) two to seven days, in 21 patients (38.9 %) eight to 14 days, and in seven patients (13 %) only a single day. Unfortunately, as only the date but not the exact time of blood sampling was documented, it was not possible to draw safe conclusions by comparing the time course of elevations of plasma glutamine (or ammonia) and outcome.

### Medication at onset of disease

In 51 patients (81 %), use of ammonia-lowering medication was documented during the initial presentation after the diagnosis of acute hyperammonemia was made. The majority of patients received infusions of L-arginine (68.3 %) and sodium benzoate (65.1 %). Other drugs were given less often, e.g. the combination of sodium phenylacetate and sodium benzoate in only 4 patients (6.3 %). A summary of all drugs given during the initial presentation is shown in Table 4.

**Table 4:** Medication given during the initial crisis

	CPS1D	OTCD	ASSD	All patients
Number of patients	12	23	27	63
Medication during initial crisis				
unclear	–	5 (21.7 %)	7 (25.9 %)	12 (19.0 %)
yes	12 (100 %)	18 (78.3 %)	20 (74.1 %)	51 (81.0 %)
L-arginine	8 (66.7 %)	15 (65.2 %)	20 (74.1 %)	43 (68.3 %)
Benzoic acid	1 (8.3 %)	–	–	1 (1.6 %)
Carglumic acid	1 (8.3 %)	3 (13.0 %)	2 (7.4 %)	6 (9.5 %)
L-citrulline	1 (8.3 %)	2 (8.7 %)	–	3 (4.8 %)
Levocarnitine	3 (25.0 %)	7 (30.4 %)	8 (29.6 %)	18 (28.6 %)
Sodium benzoate	7 (58.3 %)	15 (65.2 %)	18 (66.7 %)	41 (65.1 %)
Sodium phenylacetate & sodium benzoate	–	2 (8.7 %)	2 (7.4 %)	4 (6.3 %)
Sodium phenylbutyrate	1 (8.3 %)	4 (17.4 %)	7 (25.9 %)	12 (19.0 %)

Unclear means patients have not received medication or data were not entered

### Orthotopic liver transplantation and intake of protein

In our study population, 11 of the 51 patients surviving the neonatal period and first HE underwent OLT. Most transplantations were performed in patients with OTCD (5 of 14 OTCD patients surviving the neonatal period); two patients with CPS1D underwent OLT (2 of 9 CPS1D patients surviving the neonatal period), and four patients with ASSD (4 of 27 ASSD patients surviving the neonatal period). The indication for OLT was not recorded in this study.

**Fig. 3:**
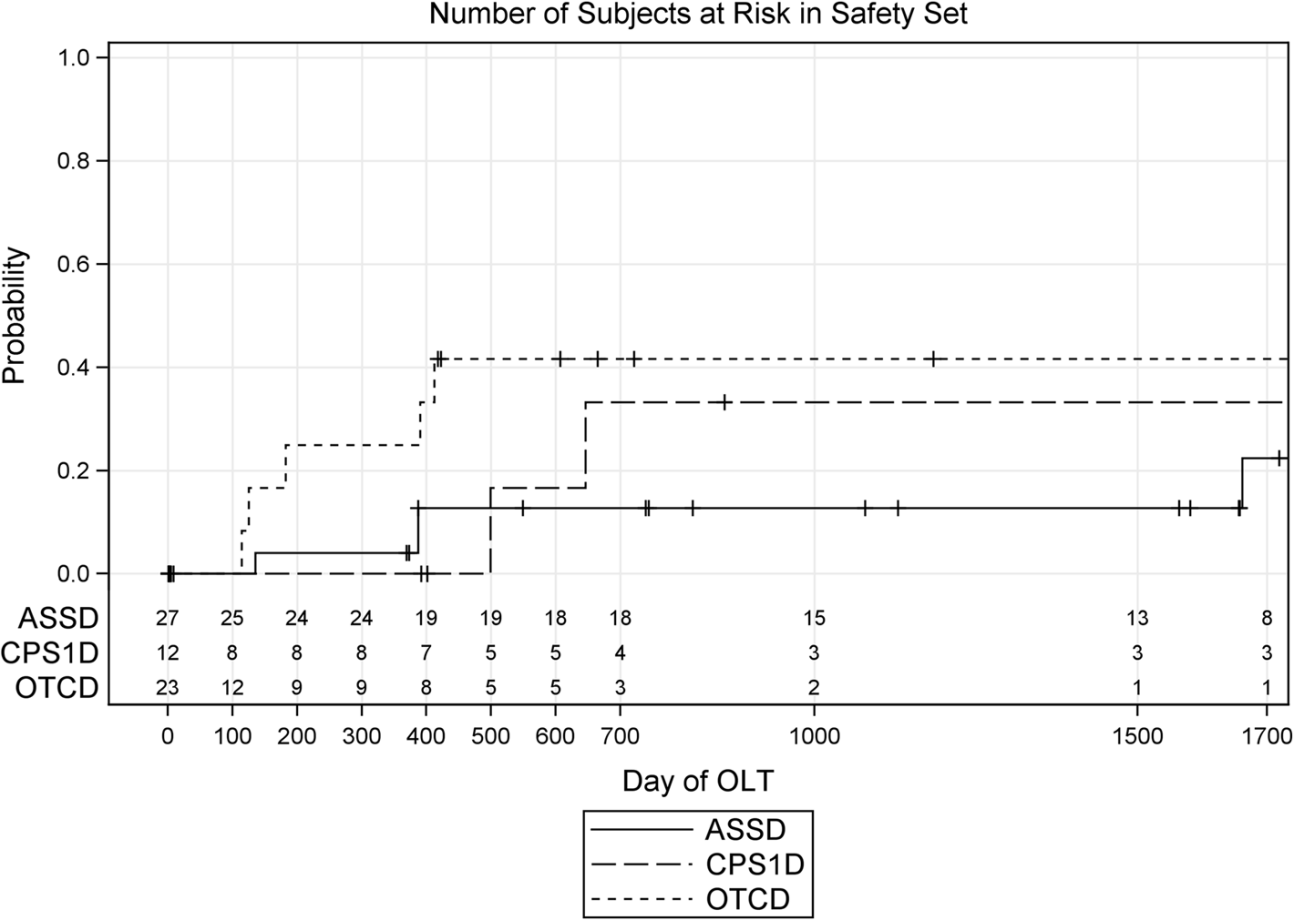
Kaplan-Meier plot of the time (shown in days of life of the patients) to orthotopic liver transplantation (OLT), separated by the three groups of patients (ASSD, CPS1D, OTCD)

Age at OLT varied widely from a minimum of 116 days to a maximum of 1663 days (mean 453 days). There was a trend that patients with OTCD were transplanted earlier if compared to patients with CPS1D and ASSD (Fig. 3).

The intake of protein in g/kg bodyweight (BW) was documented in 55 patients (26 ASSD, 19 OTCD, 10 CPS1D). In all, the intake of protein during the analysis period was restricted to 1.11 ± 0.38 g/kg BW (mean ± SD) with only marginal differences between groups: ASSD 1.25 ± 0.31, OTCD 0.99 ± 0.44, CPS1D 0.94 ± 0.35. However, the difference between the diagnosis groups with the highest (ASSD) and lowest (CPS1D) protein intake did not reach statistical significance (Kruskal Wallis test: *p* = 0.0570).

### Survival

Within the total study population of 63 patients, twelve patients (19 %) died during the initial crisis, and another four patients died within two weeks after the end of their first crisis (as already stated above, another four patients who died during their initial crisis would have also been eligible but are not included here). Thus, 16 patients (25.4 %) died during or immediately after the neonatal period. Patients with OTCD had the highest mortality rate (10 of 23 patients) followed by patients with CPS1D (4 of 12 patients), while patients with ASSD had lower mortality in this study (4 of 27 patients).

Two further deaths in patients with ASSD were reported later than 1.5 years. Thus, 28.6 % of all patients died during the total study period of five years. In addition, one patient died one day after OLT. Thus in total, 19 of the 63 patients (30.2 %) died during the observation period of five years.

### Study termination

In about half of the patients (*n* = 33; 52.4 % of total), data collection for the entire planned observation period of at least 6 months was feasible. The main reason for an early study termination was death, which occurred in 18 patients (28.6 % of total). Eleven patients (17.5 % of total) received an OLT and were only studied until this intervention. One patient was treated with liver cell infusion and was therefore removed from further analysis. Table 5 gives details of the reasons for study termination.

**Table 5:** Reason for study termination

Reason for study termination	CPS1D	OTCD	ASSD	All patients
Number of patients	12	23	27	63^a^
Death	4 (33.3 %)	10 (43.5 %)	4 (14.8 %)	18 (28.6 %)
Liver cell infusion	–	1 (4.3 %)	–	1 (1.6 %)
Orthotopic liver transplantation (OLT)	2^#^ (16.7 %)	5 (21.7 %)	4 (14.8 %)	11 (17.5 %)
Study end	6 (50.0 %)	7 (30.4 %)	19 (70.4 %)	33 ^a^ (52.4 %)

*OLT* Orthotopic liver transplantation

^a^one missing diagnosis; # one CPS1D patient died early after OLT and the cause of study termination in this patient is thus OLT

## Discussion

This was a European multicentre, retrospective, non-interventional case series reporting the natural course of neonatal onset UCD patients treated with standard care within the first five years of life. Data evaluation focussed on the basic clinical course and outcome, and on plasma levels of ammonia and glutamine at manifestation and during the course of the study. Our study is the largest of its kind in recent years in Europe, describing the natural course of 63 UCD patients treated in eight metabolic centres in Austria, France, Germany, Italy and Switzerland according to local management standards.

### Study population

There are only few case series describing the natural course after neonatal onset in UCD patients and reports from previous decades often differ in the overall management from current practice [17, 20, 23]. It is important to state that the present case series study is in line with current literature and thus comparable to other neonatal onset UCD patient series in terms of epidemiology, because ASSD was the most frequent UCD, followed by OTCD and CPS1D [24–26]; in some other reports however, OTCD was more frequent than ASSD [7, 9]. Nevertheless, the higher numbers of ASSD patients in our cohort is not in contrast to the undisputable higher incidence of OTCD as this study only includes patients with neonatal onset.

Of 190 patients screened, 63 were included in the study in accordance with the inclusion criteria. Four patients could not be included in this study, although patients would have been eligible, since the participating center did not report data. This failure to include the four patients obviously results in a bias as these patients would have increased the mortality in the early phase, or maybe even in the neonatal period.

### Survival

The mortality rate during the entire study period was 30.2 %, similar to a recent metaanalysis reporting survival of the neonatal period for 40–81 % of patients with neonatal onset UCDs, depending on their underlying defect [27]. In our study, OTCD patients had the highest death rate (43.4 %), followed by CPS1D (36.4 %) and ASSD (14.8 %). This observation is similar to more recent reports from large cohorts in the United States (US), which found mortality of neonatal onset UCD patients to be 24 % [25] and 32 % [21]. The survival data from this study are better than from reports describing patients before the year 2000, where 5-year survival rates were 22 % in patients treated before 1995 [19], 35 % in a cohort that was treated between 1975 and 1986 [20], and only 16 % in a study with 121 neonatal onset UCD patients from 1972 to 2000 [2]. In contrast, a very recent study from a multi-center investigation in the US reported a mortality rate for neonatal onset UCD patients of only 4 %, even though the size of the cohort as well as the study period were similar to the present study [26]. However, the authors mention an additional nine deceased patients that were only diagnosed postmortem and who were not enrolled in their study (which then would have resulted in a mortality rate of 11.6 %). Although by itself this only partly explains the surprisingly low mortality rate found in this study, it also points towards underreporting or sampling bias as key factors for the low rate found. Another study reporting neonatal onset UCD patients treated in the same time period in different centers in Japan also had a low mortality rate, 17 %. However, the lower mortality rate in this case may have been due to the inclusion of all UCDs [7].

It should be noted that newborn screening (NBS) for ASSD was not in place in any of the countries involved in this study. NBS may have an impact on the survival and outcome of UCD patients [28], a conclusion supported by data from the European registry, where a trend towards improved long-term neurological outcome has been found for patients with ASSD identified by NBS [29].

### Hyperammonemic events, dialysis and encephalopathy

Hemodialysis is currently the most effective way of ammonia detoxification. It is the therapy of choice if plasma ammonia levels exceed 500 μmol/L or if other measures fail to lower the ammonia concentration [6]. In line with this, 36 patients with ammonia levels > 500 μmol/L during their initial metabolic crisis received dialysis and 26 of them were in encephalopathy before dialysis was initiated. In addition, four further patients with ammonia levels > 500 μmol/L were encephalopathic during their initial metabolic crisis but did not receive dialysis. This high rate of encephalopathic patients during the first HE requiring dialysis (48 % of all patients in this study) is in line with data from Japanese centers where 59/151 (39 %) of patients were treated with dialysis during their first hyperammonemic attack (characterized by a mean peak ammonia level at onset of dialysis of 856 μmol/L) [7]. Coma is a known predictor of poor outcome [17, 18], and the vast majority of patients with an initial HE > 360 μmol/L had a poor prognosis in terms of neurodevelopmental outcome and survival despite receiving hemodialysis [7]. Thus, although our study does not provide data on neurocognitive outcome, the high ammonia levels and mortality rate in our cohort underline the need to re-evaluate current treatment strategies, including the threshold to start extracorporeal detoxification if ammonia levels exceed 500 μmol/L [6]. Because of the poor outcome in their study, Japanese authors concluded that patients with a “peak ammonia level greater than 180 μmol/l at the onset should receive hemodialysis” [7]. Such more aggressive treatment, at least of neonatal hyperammonemia exceeding 300 μmol/L, was already reported from the US where 60 % of neonatal hyperammonemic episodes, in which 50 % of neonates were “comatose”, were treated with some form of dialysis (including various methods of hemodialysis or hemo(dia)filtration, and peritoneal dialysis) [21].

### Hyperammonemic events and glutamine concentrations

Since glutamine is generally considered to be a predictor of hyperammonemic crises and of poor neurocognitive outcome [25, 30], we included this parameter in our analysis. We used as definition for significant hyperglutaminemia a plasma concentration of > 1000 μmol/L (reference ranges are age-dependent and varied slightly between study sites). During the analysis period of up to 550 days, patients suffered a mean of 2.7 episodes with a glutamine level above this limit.

### Medication at onset

Drug treatment of UCD patients always consists of a combination of nitrogen scavengers with arginine or citrulline. Depending on the specific defect, but also depending on different approaches in different countries, the details of therapy vary between centers. Despite being in use for many decades, it is still unclear whether there is a relevant difference in efficacy between these various protocols. Attempts to harmonize treatment strategies have resulted in suggested guidelines, which were based on a thorough evaluation of the literature but which were not yet in place for the patients of this study [6]. Thus, most patients in this study were treated with different medications during their metabolic crises. The most frequently used medications at onset were L-arginine (used in 68 % of patients) and sodium benzoate (used in 65 % of patients), followed by sodium phenylbutyrate or phenylacetate (used in 25 % of patients). This also reflects differences in the practice of European centers compared to those in the US, where sodium benzoate and L-arginine were only used in 5 % and 23 % of patients, respectively. Instead, the preferred initial medications in the American centers were sodium phenylbutyrate (used in 37 % of patients) and citrulline (used in 31 % of patients) [24]. The current “European” approach seems to be more in line with practices in Japan, where the majority of patients suffering from a HE received L-arginine together with sodium benzoate (e.g. used for 72 % of OTCD cases, 61 % of CPS1D cases, and 79 % of ASSD cases) [7]. This difference in the management of these most vulnerable patients, those affected by life-threatening hyperammonemia, should be seen as a chance to compare treatment approaches, something that is probably best achieved in data mining projects of the large US and European (and maybe also Japanese) UCD registries [31, 32].

### Orthotopic liver transplantation

Definitive treatment of the underlying UCD using OLT was performed in 11 patients (17.5 % of the study population; 5 OTCD, 4 ASSD, and 2 CPS1D patients). Except for two patients, this procedure was performed in the first 550 days of the study period, which likely reflects the early decision in favor of OLT although this definite treatment was done relatively late in some of the patients (mean 453 days, range 116–663). The time point of OLT in our study is in agreement with data from the US registry, which reports OLT in 42/66 patients before 2 years of age [25]. However, the number of patients receiving OLT in this study is lower than in another study from the US that reports liver transplantation in 66 % of CPS1D and OTCD patients [26]. A similar proportion of UCD patients (24 %) received liver transplantation in Japan [7]. In our cohort, one patient died one day after OLT from multiorgan failure but the numbers are too low to be compared with mortality data from other (larger) studies (in a study from Japan, one patient from a total 42 patients died of complications after the procedure [7]).

## Conclusion

This study provides data on the clinical course of 63 UCD patients with a neonatal onset of disease. The mortality rate during the entire study period of 30.2 % is higher than reported before in other recent case series. A possible explanation for this may be underreporting of severe cases in other studies. The data from this retrospective study confirm the high risk of neonatal onset UCD patients not only for an initial life-threatening decompensation but also for recurrent hyperammonemic crises, which may likewise result in permanent neurological damage. We therefore conclude that novel therapeutic approaches are still highly needed for this group of patients. Until these are effectively in place, current treatment protocols, which recommend start of extracorporeal detoxification if ammonia levels exceed 500 μmol/L, should be re-evaluated since earlier start of dialysis may be beneficial. In addition, the high risk of recurrent hyperammonemic crisis underlines recommendations to perform early liver transplantation as long as this remains the only curative approach for neonatal onset UCD patients. Finally, future studies should be planned to determine whether current patients have better outcomes than reported in this historical cohort.

## Abbreviations

ASS, argininosuccinate synthetase; ASSD, ASS deficiency; BW, bodyweight; CPS1, carbamoyl phosphate synthetase 1; CPS1D, CPS1 deficiency; HE, hyperammonemic event; NBS, newborn screening; OLT, orthotopic liver transplantation; OTC, ornithine transcarbamylase; OTCD, OTC deficiency; SELICA, Safety & Efficacy of Liver Cell Application; UCD, urea cycle disorders
